# Plasma metabolites as potential markers and targets to prevent and treat urolithiasis: a Mendelian randomization study

**DOI:** 10.3389/fmolb.2024.1426575

**Published:** 2024-08-27

**Authors:** Wuhui Zhu, Huan Li, Ming Zhang, Bing Ji, Zongtao Liu

**Affiliations:** ^1^ Qingdao Third People’s Hospital, Qingdao University, Department of Stone Disease Diagnosis and Treatment Center, Qingdao, China; ^2^ Center for Disease Control and Prevention of Shinan District, Department of Laboratory, Qingdao, China; ^3^ Integrated Traditional Chinese and Western Medicine Hospital of Shinan District, Department of Laboratory, Qingdao, China

**Keywords:** plasma metabolites, urolithiasis, Mendelian randomization, prevention, causal relationship

## Abstract

**Background:**

Studies on the relationships between diseases of the urinary system and human plasma proteomes have identified several potential biomarkers. However, none of these studies have elucidated the causal relationships between plasma proteins and urolithiasis.

**Objective:**

The objective of the study was to investigate the potential risks of plasma metabolites in urolithiasis using a two-sample Mendelian randomization (MR) study.

**Methods:**

A total of 1,400 metabolites were identified in the most comprehensive genome-wide association study (GWAS) of plasma metabolomics in a European population to date, and single-nucleotide polymorphisms (SNPs) were used as the instrumental variables for the plasma metabolites. The European GWAS data for urinary calculi included 482,123 case samples and 6,223 control samples (ebi-a-GCST90018935). The associations between the plasma metabolites and risk of urolithiasis were evaluated by inverse variance weighting (IVW) and supplemented by sensitivity analyses of the MR-Egger and MR-PRESSO tests.

**Results:**

For the first time, we found a causal relationship between two plasma metabolites (*p* < 1.03 × 10^−4^) and urolithiasis (*p* < 0.05). The chemical 4-hydroxychlorothalonil, which is an intermediate product of the pesticide hydroxychlorothalonil, could promote urolithiasis (odds ratio (OR) = 1.12) as a risk factor. Moreover, 1-stearoyl-2-arachidonoyl-GPC, which is an important component of phospholipid metabolism in the human body, can inhibit urolithiasis (OR = 0.94).

**Conclusions:**

Our results suggest that blood metabolites can be used as blood markers and drug targets in the prevention, diagnosis, and treatment of urolithiasis; furthermore, our results can provide a basis for policy makers to formulate prevention and treatment policies for urolithiasis.

## 1 Introduction

Urolithiasis is a common non-tumor disease of the urinary system. In recent decades, the incidence and prevalence of urolithiasis have increased annually, and urolithiasis has gradually become a global healthcare problem (Kittanamongkolchai et al., 2018; Scales et al., 2012). The high prevalence and recurrence rate of urolithiasis poses a heavy medical burden on society. Therefore, to prevent the formation of kidney stones, its etiology and risk factors should be considered first (Peerapen and Thongboonkerd, 2023). At present, retrospective studies are used to evaluate the risk factors of urolithiasis, but its exact etiology remains unclear. The emergence of metabolomics has provided new hope for the etiology, early diagnosis, prevention, and postoperative follow-up of urolithiasis. A recent metabolomics study based on the analysis of urine reported that succinic acid can reduce calcium deposition and damage in the kidneys, thereby inhibiting urolithiasis (Zhang et al., 2023).

At present, research on the metabolomics of urolithiasis is focused mainly on the changes in the metabolites in biological samples after stone formation. Through further analysis of differential metabolites, the associations between the metabolites and stones have been inferred. However, experiments proving the causal relationships between the metabolites and urolithiasis have not been performed because such prospective studies are often limited by ethical and time constraints, whereas retrospective studies are inevitably affected by confounding factors. Therefore, a new research method is urgently needed to prove the causal relationships between plasma metabolites and urolithiasis.

Mendelian randomization (MR) is based on the use of large samples of genetic and phenotypic data to screen single-nucleotide polymorphisms (SNPs) that have strong associations with the exposure factors and use them as instrumental variables to assess the causal relationships between the exposure and outcome factors (Gagnon et al., 2024; Xiao et al., 2022). Studies have demonstrated the causal roles of lifestyle, diseases, alcohol consumption, and other factors in urolithiasis using the MR method (Li et al., 2024; Chen T. et al., 2023). Therefore, the present study uses MR-based analyses of two samples to evaluate as well as compare the differences in the causal relationships between blood metabolites and urolithiasis so as to provide new ideas for the prevention and treatment of urolithiasis.

In this study, we used a two-sample MR design for the first time to estimate the potential causal relationships between altered plasma metabolite levels and the risk of urolithiasis. Accordingly, large-scale genetic association data were pooled (Chen Y. et al., 2023), from which the characteristics of mutation-exposure and mutation-outcome associations were derived through two independent genome-wise association studies (GWASs); these provided robust results for the causal relationships and sizes of the relationships between exposure and outcome.

## 2 Methods

### 2.1 Data

**FIGURE 1:**
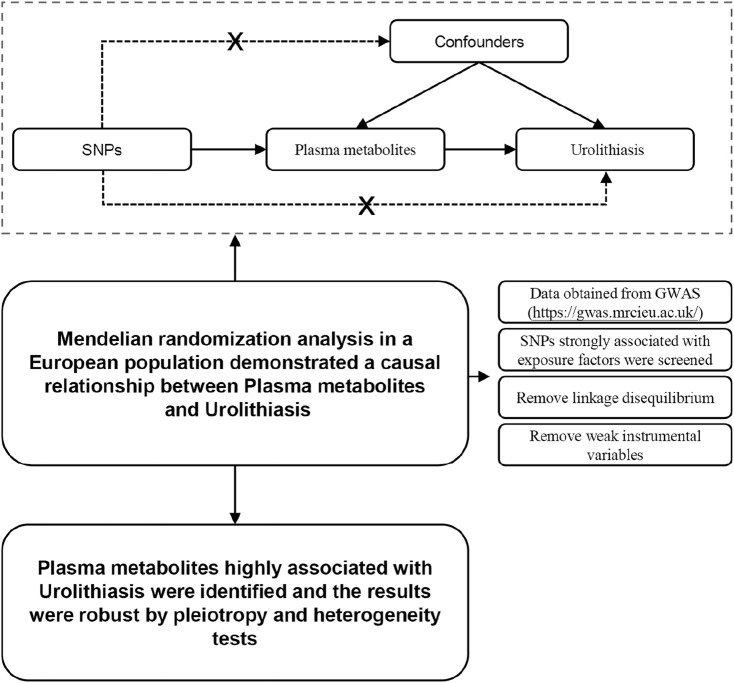
Principles and analytical procedures of Mendelian randomization.

The MR method was designed to explore the causal relationships between 1,400 blood metabolites and urolithiasis-related features using the process outlined in Figure 1. The study was conducted at the Third People’s Hospital of Qingdao, Shandong Province, China and completed in April 2024. Two datasets collected by Chen Y. et al. (2023) containing data on 1,091 metabolites and 309 metabolites through GWASs were used here as these are by far the most comprehensive analyses of blood metabolites. Genetic signals from known genes with strong biological confidence in terms of the metabolites were used to infer the causal associations between metabolite levels and factors for 12 traits and diseases that were primarily influenced by different mechanisms (such as aging, metabolism, and immune responses) in a cohort of 8,299 adults of European origin (details are provided in Supplementary Material S1). The GWAS analysis data of urinary calculi were obtained from the IEU Open GWAS project (http://mrcieu.ac.uk), which included 482,123 case samples and 6,223 control samples (ebi-a-GCST90018935) (Sakaue et al., 2021). No ethical review was required here because the study data were derived from public databases.

### 2.2 Selection of instrumental variables

The instrumental variables used in the MR analyses should satisfy three criteria (Evans and Davey, 2015; Didelez and Sheehan, 2007). First, the instrumental variables should be related to the exposure; second, the instrumental variables should be independent of the outcomes (urolithiasis-related characteristics); third, the instrumental variables should not affect the results in ways other than exposure. To satisfy the first condition, the genome-wide significance threshold of the blood metabolites was set at *p* < 1.00 × 10^–5^, and the linkage disequilibrium (LD) thresholds were set at r^2^ = 0.001 and kb = 10,000 to select independent instrumental variables. The F-values of the SNPs were calculated for the obtained instrumental variables. To exclude weak instrumental variables, only those variables with F-values >10 were finally included in the MR analyses (Yu et al., 2022). Thus, the 1,400 metabolites finally selected from the data sources had strongly associated SNPs, with the LD and weak instrumental variables removed.

### 2.3 Mendelian randomization

The two-sample MR analysis was used in this study and five methods were implemented, namely the MR-Egger, weighted median, inverse variance weighted (IVW), simple mode, and weighted mode methods. The IVW method used as the main analysis approach estimates the causal effects of the genes on the traits by weighting the causal effects of different genetic variants on the traits and then combining the estimated effects after weighting. The advantages of the IVW method include reduced influence of the sample size, improved estimation accuracy, and reduced bias, which were the primary reasons for its use as the main analysis method (Sanderson, 2021). The MR-Egger and weighted median methods were used as supplementary approaches, and the β value was calculated to determine the consistency of the results to enhance the robustness to causality. The MR-Egger method can be used to calculate the direct and indirect effects as well as evaluate multiple effects of the genetic variations on the results so as to adjust the confounding bias while improving the accuracy of causality estimation to a certain extent (Burgess and Thompson, 2017). The weighted median method mainly assigns different weights to different genetic variants, thereby reducing the impacts of extreme genetic variation on causal inference and improving the stability of the results. In addition, the weighted median method has the advantages of improved estimation accuracy, wide applicability, and strong flexibility (Hartley et al., 2022).

### 2.4 Screening of metabolites associated with stones

As noted above, five methods were used for MR. Among the 1,400 metabolites, those satisfying both *p* < 0.05 and false discovery rate (FDR) < 0.2 were associated with urolithiasis. The results of the metabolites associated with urolithiasis after screening are shown in Supplementary Material S4, and pleiotropic tests of the 1,400 metabolites are shown in Supplementary Material S5.

### 2.5 Robustness of the results

The heterogeneity of the effects of blood-metabolism-related SNPs on urolithiasis outcome was evaluated using Cochran’s Q test, for which there was no significant heterogeneity among the SNPs when *p* ≥ 0.05. Otherwise, there was heterogeneity, and the random effects model was applied to the IVW method to estimate causality. MR-PRESSO regression was used to determine if there was horizontal pleiotropy; when *p* < 0.05, horizontal pleiotropy was considered to exist. Finally, the sensitivity of the results was tested by the leave-one-out method, where the SNPs were individually eliminated to determine whether each of them had an impact on the estimated value from the IVW method. If the results changed significantly after excluding an SNP, it was considered that the MR results were sensitive to that SNP. When urolithiasis was used as the outcome, the β value and 95% confidence interval (CI) were used if the effect size was a continuous variable. If the effect size was a dichotomous variable, it was expressed in terms of the odds ratio (OR) and 95% CI. We used R software version 4.3.1 for the above analyses. To control the false positive error rate, the *p*-value threshold was adjusted by the FDR to determine causality, and metabolites with FDR < 0.2 were considered to have significant causal relationships (Wang et al., 2023).

## 3 Results

### 3.1 Instrumental variable screening

Based on the genome-wide significance and LD, 1,400 blood metabolites were selected as the eligible instrumental variables. The number of instrumental variables for each metabolite ranged from 11 to 86. The minimum and maximum F-values of the SNPs of the 1,400 blood metabolites were 19.50 and 5,308.35, respectively, with both meeting the F > 10 requirement. This suggests that the study was less likely to be affected by weak instrumental variables, and the results are shown in Supplementary Material S2.

### 3.2 MR analysis

**FIGURE 2:**
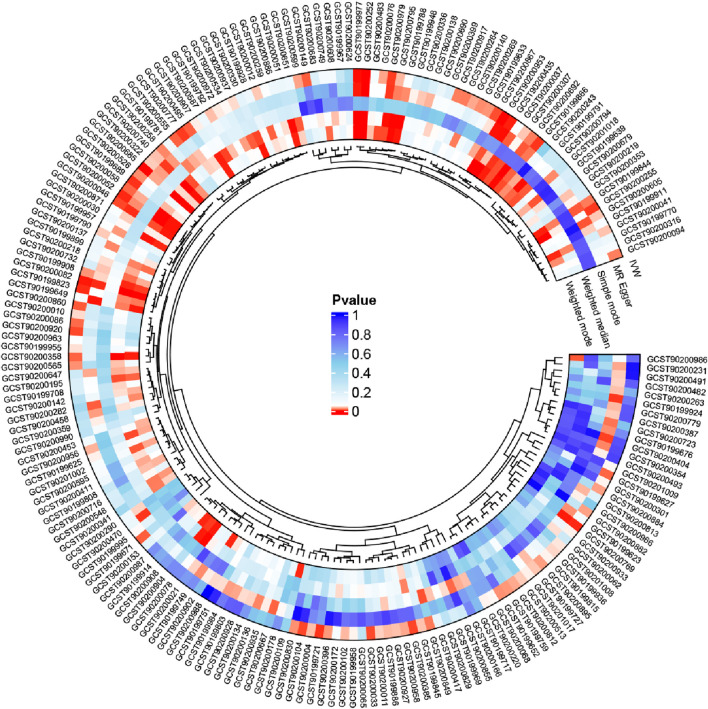
Five methods were used for Mendelian randomization in this study, and the results are represented in the form of the circle diagram. The red-colored data indicate *p* < 0.05. The metabolites shown in red color are the risk factors associated with urolithiasis based on the inverse variance weighting (IVW) method.

Five MR methods were used to evaluate the causal effects of the 1,400 blood metabolites on urolithiasis. Metabolites with *p*-value <0.05 obtained by any method were shown in the result matrix that was represented by a circle plot. The red-colored data in the circle diagram indicate those with *p*-values <0.05, and the darker the color, the closer is the *p*-value to 0. The darker blue colors indicate *p*-values closer to 1, and the metabolites in red represent the risk factors associated with urolithiasis (Figure 2; Supplementary Material S3).

### 3.3 Screening of metabolites

**FIGURE 3:**
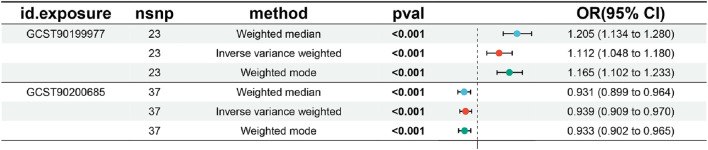
Mendelian randomization analysis shows that two metabolites have causal relationships with urolithiasis (*p* < 0.05). The odds ratio (OR) value of 1-stearoyl-2-arachidonoyl-GPC was 0.94 by the IVW method, indicating that it is a protective factor in urolithiasis. The OR value of 4-hydroxychlorothalonil was 1.12 by the IVW method, indicating that it was a risk factor in urolithiasis.

A total of 90 metabolites that had *p*-values <0.05 from the results of the IVW method were used in the main MR method (Supplementary Material S5). However, there were only two metabolites with *p*-values <0.05 from the five MR methods, indicating that only these two metabolites (1-stearoyl-2-arachidonoyl-GPC and 4-hydroxychlorothalonil) had strong causal relationships with urolithiasis (Figure 3; Supplementary Material S6). The OR value of 1-stearoyl-2-arachidonoyl-GPC was 0.94 by the IVW method, indicating that 1-stearoyl-2-arachidonoyl-GPC is a protective factor in urolithiasis. The OR value of 4-hydroxychlorothalonil was 1.12 by the IVW method, indicating that 4-hydroxychlorothalonil was a risk factor in urolithiasis.

### 3.4 Robustness analysis

**FIGURE 4:**
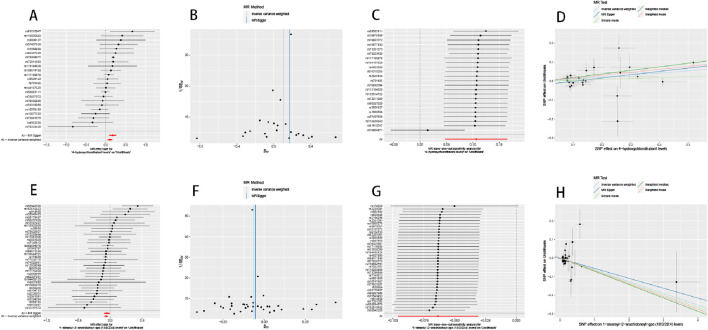
Mendelian randomization analysis between plasma metabolites and urolithiasis. **(A, E)** Forest plots of Mendelian randomization for the two identified plasma metabolites, showing the effect and total effect (β) values of each single-nucleotide polymorphism (SNP). **(B, F)** Funnel plots to detect whether the results of the two plasma metabolites are biased. **(C, G)** Sensitivities of the leave-one-out method for analyzing the SNPs of the two plasma metabolites. After removing each SNP, the results of the remaining SNPs were analyzed and shown to remain robust. **(D, H)** Scatter plots of the five analytical methods used for Mendelian randomization. The abscissa shows the effects of the SNPs on the exposure factors, and the ordinate shows the effects of the SNPs on the outcomes.

The results of the MR of metabolites in urolithiasis were further analyzed for robustness. There was no significant heterogeneity between the SNPs (Supplementary Material S7, S8) based on Cochran’s Q test with *p* ≥ 0.05. The MR-PRESSO test showed that there was no horizontal pleiotropy in the causal relationships between urolithiasis and 1-stearoyl-2-arachidonoyl-GPC as well as 4-hydroxychlorothalonil (Supplementary Material S9, S10). The leave-one-out method showed that the causal relationships were stable after eliminating the SNPs individually. The corresponding findings are depicted via funnel and forest plots in Figure 4.

## 4 Discussion

In recent years, urolithiasis has been recognized as a metabolism-related disease because it coexists with various metabolic disorders and because its related metabolites and metabolic pathways have been found. This study identified 90 metabolites with potential causal relationships with urolithiasis-related features, among which 4-hydroxychlorothalonil and 1-stearoyl-2-arachidonoyl-GPC had Bonferroni-corrected significance for urolithiasis, indicating robust causal relationships based on the sensitivity analysis. The results showed that 4-hydroxychlorothalonil could promote urolithiasis (OR = 1.12) while 1-stearoyl-2-arachidonoyl-GPC had a protective effect on urolithiasis (OR = 0.94). This study reveals for the first time that genetic inheritance could provide a new perspective on the pathogenesis of urolithiasis as well as potential directions for precise treatment.

Li et al. (2024) used mediation MR and found that the plasma metabolites phenylalanine, XL_VLDL_PL_pct mediated the causal relationships between lifestyle habits (smoking, drinking, and tea consumption) and kidney stones. Compared with our study, their datasets of the exposure factors and outcomes were different, resulting in screening of different metabolites. Another study by Chen T. et al. (2023) showed that the levels of blood sugar (OR = 1.002), maltotriose (OR = 0.998), and X-24947 (OR = 0.999) had causal effects on kidney calculi. Chen T. et al. (2023) used the same plasma metabolite dataset as that used in our study, but their outcome dataset was different. In addition, the results of our study are more rigorous and reliable because the *p*-values of all five MR results were under 0.05. However, compared with the study by Chen T. et al. (2023), the study by Li et al. (2024) had different exposure factor and outcome datasets, so the final metabolites screened were also different. The above results indicate that more studies using different datasets are needed to analyze and validate the causal relationships between plasma metabolites and urolithiasis.

The results of the present study suggest a causal relationship between 4-hydroxychlorothalonil and urolithiasis; 4-hydroxychlorothalonil is a degradation product of chlorothalonil that may be more toxic and environmentally persistent than chlorothalonil, which could have toxic effects on aquatic organisms (Wu et al., 2014; Xu et al., 2020; Krais et al., 2023). The substance 4-hydroxychlorothalonil has recently been identified in human serum and breast milk; it was found in 1,808 maternal serum samples from the general population in Sweden (1997–2015) and in 393 maternal serum samples from the agricultural population in Costa Rica (2010–2011). The median concentrations of 4-hydroxychlorothalonil were 4.1 μg/L in the Swedish and 16.1 μg/L in the Costa Rican (3.9-fold higher) populations, and women living in the tropical agricultural conditions of Costa Rica had higher serum chlorothalonil concentrations than the Swedish general population (Krais et al., 2023). Recent studies from China have shown that 4-hydroxychlorothalonil is present in the breast milk and sera of Chinese people from different regions (Zhang et al., 2024). It has been shown that 4-hydroxychlorothalonil enhances the production of proinflammatory cytokines in the keratinocytes and that exposure to 4-hydroxychlorothalonil in the environment could increase the risk of inflammatory skin diseases in humans (Xu et al., 2020). Another study found that 4-hydroxychlorothalonil has a strong endocrine disrupting effect on zebrafish (Zhang et al., 2016). In addition, a latest MR study has found a causal relationship between 4-hydroxychlorothalonil and tumors of the urinary system, revealing potential biomarkers and therapeutic targets for urinary system tumors (Dai et al., 2024). Because studies on the effects of 4-hydroxychlorothalonil on urinary diseases are very limited, the results of the present study suggest that 4-hydroxychlorothalonil may act through the neuroendocrine–immune circuit, which needs to be verified through further experiments. Our study found for the first time that the plasma metabolite 4-hydroxychlorothalonil plays an important role in urolithiasis, and our results indicate that 4-hydroxychlorothalonil is a potential risk factor, marker, and therapeutic target for urolithiasis. At the same time, the results suggest that the government and society should pay more attention to controlling 4-hydroxychlorothalonil pollution of the soil and water to avoid health hazards to the populations in the corresponding areas.

Our study found that 1-stearoyl-2-arachidonoyl-GPC (18:0/20:4) has a causal relationship with urolithiasis and can inhibit it. Glyceryl phosphoryl choline (GPC) is very important for maintaining the stability of cell membrane. Studies have found that GPC is closely related to the occurrences of cardiovascular and cerebrovascular diseases, liver diseases, and cognitive function disorders (Lee et al., 2021; Lee et al., 2017; Tayebati and Amenta, 2013; Hirabayashi et al., 2023; Bozelli and Epand, 2019; Muta et al., 2022), whereas 1-stearoyl-2-arachidonoyl could cause cancer by mediating lipid metabolism imbalance (Bozelli and Epand, 2019). Recent studies have shown that 1-stearoyl-2-arachidonoyl-GPC has a protective effect on inflammatory bowel disease (Di’Narzo et al., 2022). Another recent MR study showed that 1-stearoyl-2-arachidonoyl-GPC could promote the development of allergic rhinitis (Chen et al., 2024). Thus far, there are no reported studies on the role of 1-stearoyl-2-arachidonoyl-GPC in urolithiasis. It is possible that 1-stearoyl-2-arachidonoyl-GPC could play a role in urolithiasis by regulating lipid metabolism and inflammation, but this hypothesis needs to be confirmed by experiments.

Recent studies have shown that the ratio of glucose to mannose is particularly high in *C. militaris* fruiting bodies and has protective effects against inflammatory bowel disease; this suggests that the ratio of glucose to mannose could be an indicator of inflammation inhibition (Guo et al., 2024). According to the table in Supplementary Material S3 and based on calculations by the IVW method, the *p*-value of 3,5-dichloro-2,6-dihydroxybenzoic acid and its glucose-to-mannose ratio were higher than those of 1-stearoyl-2-arachidonoyl-GPC and 4-hydroxychlorothalonil; hence, these two substances are potential metabolites with causal effects on urolithiasis. In addition, 3,5-dichloro-2,6-dihydroxybenzoic acid was found to improve organ metabolic function in a metabolomics analysis study for the treatment of hypertrophic cardiomyopathy (Wolter et al., 2021). Since the two identified metabolites may have protective effects in inflammatory bowel disease as well as myocardial disease and since our findings suggest that they could promote urolithiasis in the absence of sufficient literature to provide more information, further experiments are needed to explore and validate these observations.

The present study has certain prominent limitations. First, we need to validate our MR results using metabolomics to explore the differences in blood metabolites in non-European ethnic groups; second, the validity of the MR analysis depends heavily on interpretation of the instrumental variables of exposure, and it is necessary to expand the sample size to more accurately assess the genetic influences on blood metabolites. Most importantly, in the case of datasets in public databases, only the race and sample size of the data source are known, while specific details from the grouping population, such as the compositions of the stones, are not known. Because different metabolites can play different roles in the formation of stones with different compositions, if we use MR methods on datasets containing information on the stone composition, then the resulting metabolites with causal effects are expected to be more accurate.

In conclusion, using two-sample MR analysis, two plasma metabolites, namely 4-hydroxychlorothalonil and 1-stearoyl-2-arachidonoyl-GPC, were observed to have promoting and inhibiting effects on urolithiasis, respectively. The results of this study contribute to a deeper understanding of the genetic relationships between blood metabolites and urolithiasis; moreover, the blood metabolite 1-stearoyl-2-arachidonoyl-GPC could be used as a potential biomarker in the exploration of targeted drugs for the treatment of urolithiasis. More importantly, from the perspective of public health, this study provides an important basis for policy makers and stakeholders in the government to improve awareness and formulate policies regarding 4-hydroxychlorothalonil contamination of soil and water that could cause stone disease.

## Data Availability

The original contributions presented in this study are included in the article/Supplementary Material, further inquiries can be directed to the corresponding authors.
